# An approach of identifying differential nucleosome regions in multiple samples

**DOI:** 10.1186/s12864-017-3541-9

**Published:** 2017-02-07

**Authors:** Lingjie Liu, Jianming Xie, Xiao Sun, Kun Luo, Zhaohui Steve Qin, Hongde Liu

**Affiliations:** 1 State Key Laboratory of Bioelectronics, School of Biological Science & Medical Engineering, Southeast University, Nanjing, 210096 China; 2 Department of Neurosurgery, Xinjiang Evidence-Based Medicine Research Institute, First Affiliated Hospital of Xinjiang Medical University, Urumqi, 830054 China; 3 Department of Biostatistics and Bioinformatics, Rollins School of Public Health, Emory University, Atlanta, GA 30322 USA

**Keywords:** Nucleosome, Multiple cell types, Differential nucleosome regions (DNRs), Chi-squared test

## Abstract

**Background:**

Nucleosome plays a role in transcriptional regulation through occluding the binding of proteins to DNA sites. Nucleosome occupancy varies among different cell types. Identification of such variation will help to understand regulation mechanism. The previous researches focused on the methods for two-sample comparison. However, a multiple-sample comparison (*n* ≥ 3) is necessary, especially in studying development and cancer.

**﻿Methods:**

Here, we proposed a Chi-squared test-based approach, named as Dimnp, to identify differential nucleosome regions (DNRs) in multiple samples. Dimnp is designed for sequenced reads data and includes the modules of both calling nucleosome occupancy and identifying DNRs.

**Results:**

We validated Dimnp on dataset of the mutant strains in which the modifiable histone residues are mutated into alanine in *Saccharomyces cerevisiae*. Dimnp shows a good capacity (area under the curve > 0.87) compared with the manually identified DNRs. Just by one time, Dimnp is able to identify all the DNRs identified by two-sample method Danpos. Under a deviation of 40 bp, the matched DNRs are above 60% between Dimnp and Danpos. With Dimnp, we found that promoters and telomeres are highly dynamic upon mutating the modifiable histone residues.

**Conclusions:**

We developed a tool of identifying the DNRs in multiple samples and cell types. The tool can be applied in studying nucleosome variation in gradual change in development and cancer.

**Electronic supplementary material:**

The online version of this article (doi:10.1186/s12864-017-3541-9) contains supplementary material, which is available to authorized users.

## Background

Nucleosome is basic unit of eukaryotic chromatin and consists of 147 bp DNA wrapping around a histone octamer core [1]. The nucleosomes can inhibit the access of the protein binding to the nucleosomal DNA, thus having roles in transcription regulation [2]. Nucleosome organization is highly dynamic during differentiation, extracellular stimulus and tumorigenesis [3–6]. It is important to identify such dynamics in interpreting the regulation mechanism in which the nucleosomes involve. By the virtue of high throughout sequencing techniques, we can map the nucleosomal DNA and infer the positions and occupancies of the nucleosomes. For two-sample comparison, methods and tools of identifying differential nucleosome regions (DNRs) are available now. Chen et al. developed a pipeline Danpos to call the differential signal [7]. In Danpos, when nucleosome occupancy is higher in sample A than in sample B, *P*-value of nucleosome occupancy in sample A is calculated based on Poisson distribution with lambda defined by nucleosome occupancy in sample B. Fu et al. developed a approach with non-parametric test (K-S test) to find the differential nucleosome positioning [8]. A sampling strategy is used to control the false discovery ratio. Jason et al. compared the nucleosome occupancy using a window-based negative binomial distribution method and found the nucleosomes were altered at enhancers during cell differentiation [9]. Another method of identifying two-sample differential signal includes two steps: peak finding and peak comparison [10]. But its validity on nucleosome dataset is unknown. Mahony S. et al. developed a generalized Expectation Maximization-based model MultiGPS to detect differential transcription factor binding enrichment across multiple experimental conditions [11].

Nucleosome occupancy varies in a continuous manner during differentiation, tumorigenesis and response for stimulus from outsides [4, 12, 13]. In order to resolve the roles of nucleosomes, more of samples should be taken in comparison. This requires a method that is able to identify the DNRs in more than two samples (or cell types), namely a method for multiple-sample comparison. The two-sample methods are not suitable for this situation any more.

In this paper, we described an approach (Dimnp) that can be used to identify the DNRs in multiple samples. We showed that Dimnp is able to call the DNRs for both two samples and more than three samples. With Dimnp, we revealed that nucleosomes are highly dynamic at promoters and telomeres upon mutating the modifiable histone residues.

## Methods

### Dataset

In order to resolve the nucleosome variation in response to the mutation on the individual modifiable histone residue, we determined the nucleosome occupancy in twenty two histones mutants in *Saccharomyces cerevisiae* [6]. In those mutants, the modifiable lysine (K) and arginine (R) of four core histones were respectively substituted with the non-modifiable residue alanine (A). In the present study, the dataset is used to validate Dimnp. The dataset of nucleosome occupancy is available at website http://bioinfo.seu.edu.cn/Nu_dynamics_data_public/.

### Identification of the differential nucleosome regions

Our approach has three steps, including calculating nucleosome occupancy, calling differential significance (*P*-value) and identifying the DNRs. In the first step, with mapped reads data, nucleosome occupancy is estimated and normalized for each sample so that the resulting signal is suitable for comparison. Firstly, the mapped reads are extended to 75 bp in 3′ direction and then shifted 37 bp to 3′ direction. Secondly, nucleosome occupancy profile is calculated by summating the reads count at each genomic locus. Thirdly, the profile is normalized by dividing it by the average reads count of the whole chromosome, i.e. a global background correction. Through the procedure, the nucleosome occupancy profile (reads count) of each locus is represented with a fold change to the average reads count, which eliminates the effect caused by different sequencing depth in the samples. We also provided an option to perform a local background correction, in which the profile is normalized by dividing the profile by the average of reads count of a 10-kbp sliding window at each genomic locus.

In the second step, a Chi-squared test-based model is applied to identify the DNRs. For *n* samples, given *a*_*i*_, representing the normalized reads count at locus *k* in sample *i*, and *b*_*i*_, representing the background of the normalized reads count at locus *k* in sample *i*, the test statistic χ^*2*^ at locus *k* is calculated with Eq. 1.

1 $$ {\chi}^2={\displaystyle \sum_{i=1}^n\left[\frac{{\left({a}_i-{A}_i\right)}^2}{A_i}+\frac{{\left({b}_i-{B}_i\right)}^2}{B_i}\right]}, v= n-1; i=1,\ 2, \dots, n, $$

Here, *A*_*i*_ and *B*_*i*_ represent the expected values for *a*_*i*_ and *b*_*i*_, respectively. The background *b*_*i*_ is an average normalized reads count of the chromosome. Then, significance *P*-value is calculated using Eq. 2.

2 $$ \mathrm{P}\hbox{-} \mathrm{value}=1- chicdf\left({\chi}^2, n-1\right) $$

Here, *chicdf* represents the Chi-square cumulative distribution for χ^2^ with the degree of freedom *n*-1.

In the third step, the DNRs are identified. Firstly, given a cutoff for the *P*-values, the regions with a *P*-value less than the cutoff are regarded as candidate DNRs. Then the candidate DNRs less than 10 bp are neglected, and any two adjacent DNRs with a distance less than 5 bp are merged into one DNR. We named the method with Dimnp, representing “differential identification in multiple (*n* ≥ 3) samples”.

### Validation of Dimnp

In order to test the capacity of Dimnp, we manually identified the DNRs in three cell types, and compared them with the DNRs identified by Dimnp. The three cell types include wild type (H4WT), mutants H4R3A (arginine (R) 3 of histone H4 was mutated into alanine (A)) and H4K20A. In manual identification, we firstly performed a pairwise comparison by calculating a ratio of the normalized reads counts in any two samples. Those regions with a ratio of more than 1/0.6 or less than 0.6 are defined as DNRs. Then, we pooled the pairwise comparison DNRs together, forming the DNRs data for the three cell types. We marked the DNRs as “the manual”. Actually, the manual identification is a computational algorithm. At last, receiver operating characteristic (ROC) curves are employed in evaluating the performance. If a DNR by Dimnp overlaps a DNR by the manual, the former DNR is regarded as a positive DNR.

We also called the DNRs with literature tool Danpos in two cell types with default settings except parameter “-t 10^−5^” (*P*-value cutoff). By pooling the two-cell type DNRs together, we generated the DNRs data for the multiple cell types and compared them with the DNRs identified by Dimnp. A matching percentage was used to evaluate the extent of the matching DNRs between Dimnp and Danpos. The matching percentage is a ratio of the number of matched DNRs between Dimnp and Danpos relative to the total number of the DNRs by Dimnp under a specified deviation. The deviation is from 1 bp to 100 bp.

### Software package of Dimnp

We developed two packages for Dimnp (Additional file 1). One is based on Python (version 2.7) and for Linux operation environment. This package needs the third-party Python package rpy2 and R environment (version 3.0.2). The other is written with Matlab (R2009) and can be used in Windows operating systems. The input file is the mapped reads data in bowtie or bed format. The Python-based package contains two modules: *NuclPreprocess.py* and *CalDiffPoints.py*. Function *NuclPreprocess.py* is used to calculate normalized reads count for each locus of genome. Usage: NuclPreprocess.py [−h] [−o output_path] [−f (bed, bowtie)] [−p paired] input_path. For argument -p, 1 is for paired-end reads data and 0 for single-end reads data. Function *CalDiffPoints.py* is used to call the DNRs. Usage: *CalDiffPoints.py* [−h] [−c CUTOFF] [−o output_path] file_names [file_name …]. Argument -c is for setting cutoff for *P*-value. In Matlab language (R2009), the core function of Dimnp is *Statistical*_*test*_*forN.m*. Usage: [*Pval*_*lg*, *FDR*_*cutoff*, *region*_*filtered*] = Statistical_test_forN (*file*_*out*, *cutoff*, *N*_*flag*, *varargin*), where *varargin* are the variables of nucleosome occupancy profile, *cutoff* is *P*-value cutoff and *N*_*flag* indicates the normalization way. Information of the DNRs (*region*_*filtered*) will be written into a file (*file*_*out*). It also estimates the false discovery ratio (FDR) for each DNR.

Both the program (Additional file 1) and the dataset are available at website: http://bioinfo.seu.edu.cn/Nu_dynamics_data_public/.

An enrichment analysis was carried out for nucleosome-dynamic genes with tool DAVID (http://david.abcc.ncifcrf.gov/).

## Results

### Performance of Dimnp

In order to test the quality of the sequenced dataset, we aligned nucleosome occupancy profile at transcription start sites (TSSs) for 5419 yeast genes (Additional file 2: Figure S1). The profiles show a typical nucleosome depleted region near TSSs and a well positioned nucleosome array downstream of TSSs, which indicates a high quality of the dataset.

**Fig. 1:**
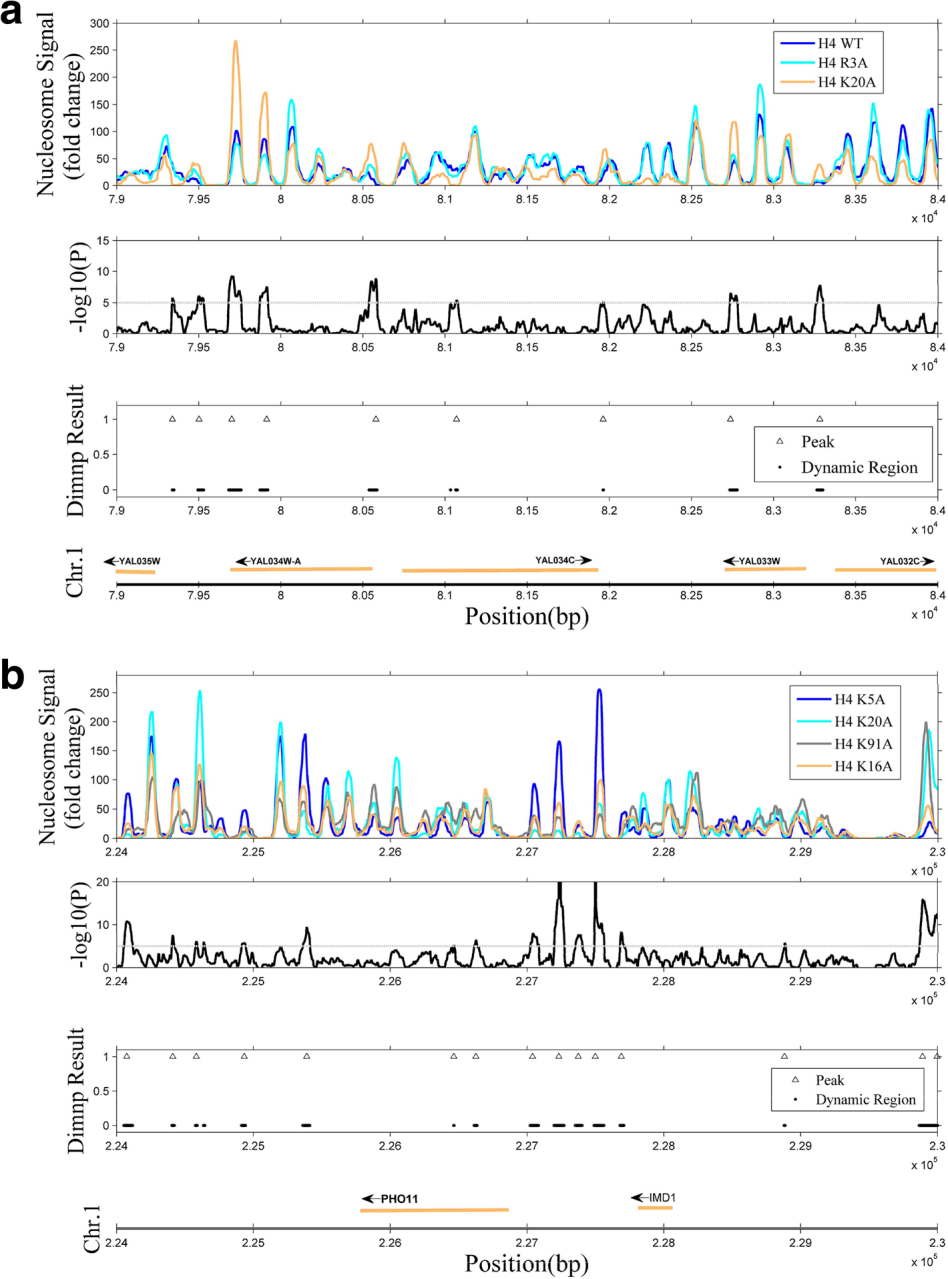
Identification of the differential nucleosome regions (DNRs) in multiple cell types. Shown are samples of the identification in three cell types (**a**) and four cell types (**b**). In each subplot, the normalized reads count (nucleosome occupancy) is shown at the top panel. The *P*-value for each genomic locus is in the middle panel. The *P*-value cutoff is 10^−5^ (dot line). The third panel shows the DNRs (dot) and the DNRs’ center position (triangle). The gene regions are marked at the bottom panel. Subplot A is for wild type (H4WT) and mutants H4R3A and H4K20A. Subplot B is for four mutants H4K5A, H4K20A, H4K91A and H4K16A

Figure 1 shows Dimnp identification across multiple cell types. Figure 1a is for three cell types, wild type (H4WT), H4R3A and H4K20A; Fig. 1b is for four cell types, H4K5A, H4K20A, H4K91A and H4K16A. In each subplot of Fig. 1, the top panel shows the nucleosome occupancy profiles. The second panel indicates the *P*-values at each locus. In the third panel, dot lines indicate the DNRs and the “△” indicates the center positions of the DNRs. As shown in Fig. 1, those regions with small *P*-values (≤10^−5^) exactly correspond to the regions where the nucleosome occupancy greatly varies across the cell types. Correspondingly, a region with a great *P*-value shows a less change in the occupancy. This indicates that Dimnp is sensitive to the difference of the normalized reads count across the cell types. By setting a *P*-value cutoff, it is easy to identify the DNRs. For each DNR, Dimnp calculates the boundary, the center position, *P*-value and false discovery ratio (FDR) (Additional file 3) (Fig. 1). This result indicates that Dimnp is feasible in identifying DNRs across multiple cell types.

**Fig. 2:**
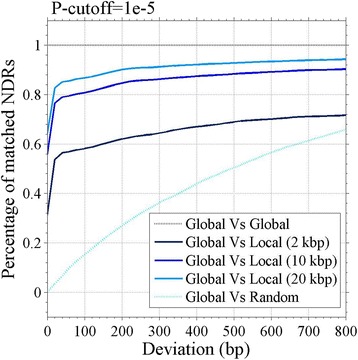
Comparison of local and global background correction methods in Dimnp. Shown is the matching percentage between two correction methods for cell types H4WT, H3R4A and H4K20A (*P*-value cutoff = 10^−5^). The DNRs were identified with the local and global background correction methods, respectively. The matching percentage (y-axis) is the ratio of the matched DNRs number between the two methods (the global and the global) relative to the total number of DNRs with the global method under a certain deviation (from 1 bp to 800 bp (x-axis))

Normalization is critically important to DNR identification. We compared the effect of global and local background correction methods (Fig. 2, Additional file 2: Figure S2). The result indicates that the local correction with a window of more than 10 kbp has a similar effect with the global correction. A small window magnifies the noise and represses the signal of nucleosome (Additional file 2: Figure S3) and will result in more false positive.

**Fig. 3:**
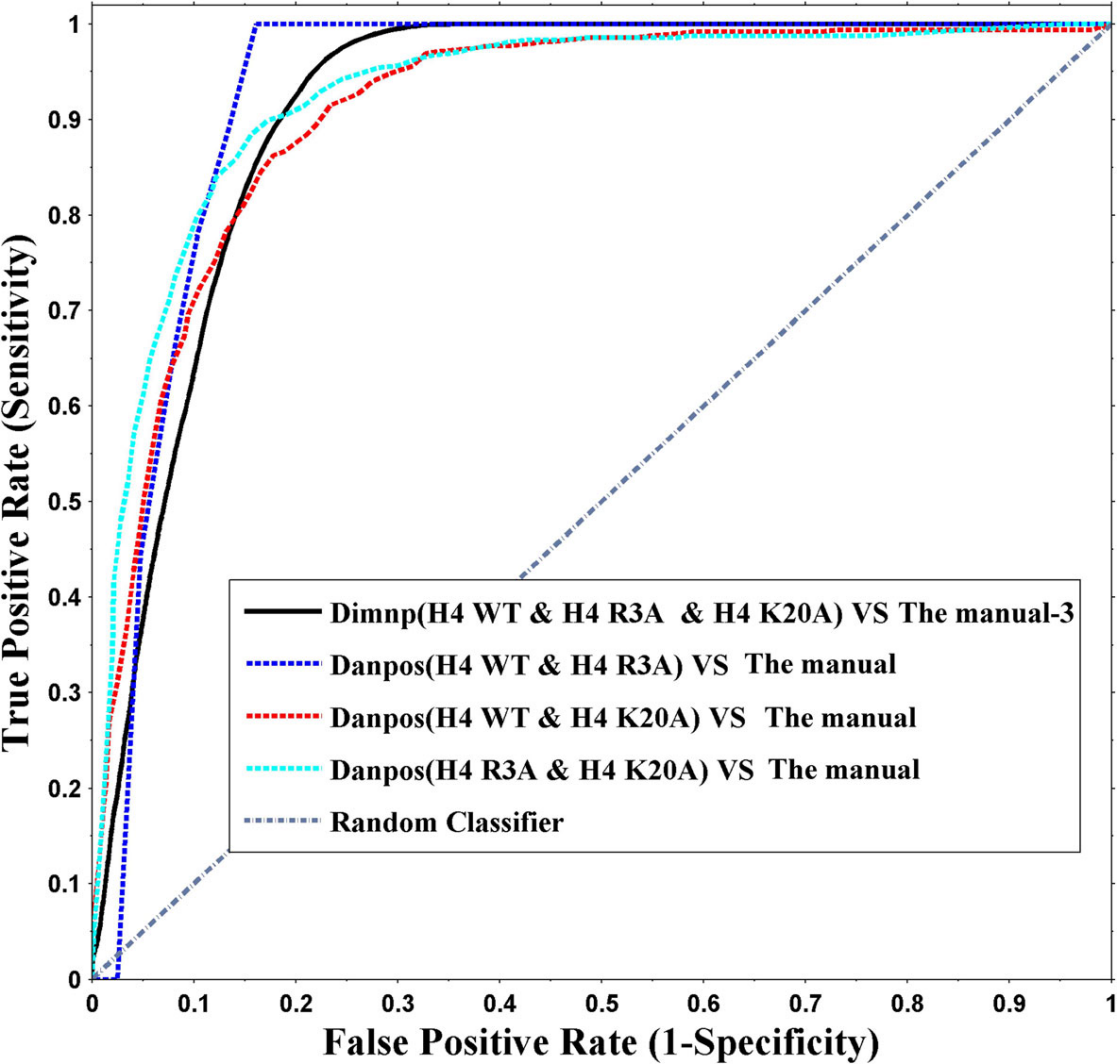
Validation for Dimnp using the manually identified DNRs as a standard. Shown are receiver operating characteristic (ROC) curves in comparing the DNRs by Dimnp and the manually identified DNRs. The manual DNRs between two cell types, termed as “the manual”, are the regions where the ratio of the normalized reads count between the two cell types is either ≥1/0.6 or ≤ 0.6. The manual DNRs of three cell types (“the manual-3”) are formed by polling the manual two cell types DNRs together. The pooled manual DNRs are used as a standard to test the performance of both Dimnp and literature tool Danpos [7]. The area under the curve (AUC) is 0.87 for Dimnp

In order to validate our model Dimnp, we manually identified the DNRs among WT, H4R3A and H4K20A (see Method). Using the manual identification result as a standard, we calculated the ROC curves for Dimnp and the literature tool Danpos, respectively (Fig. 3). The ROC curve indicates a good matching between the manual identification (termed as “the manual-3”) and the Dimnp identification (area under the curve (AUC) =0.87). Importantly, Dimnp is able to capture all the DNRs in one-time calculation for the three cell types. Because Danpos is good at two-cell type comparison, it shows a AUC of more than 0.89 in comparing with the two-cell type manual DNRs (Fig. 2), indicating a good performance.

**Fig. 4:**
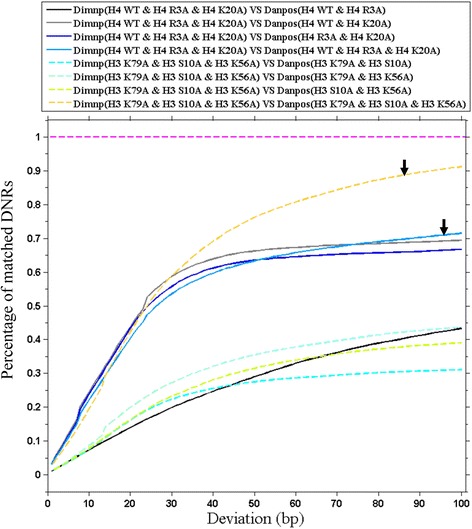
Percentage of the matched DNRs between Dimnp and Danpos. The percentage (vertical axis) represents the ratio of the number of matched DNRs between Dimnp and Danpos relative to the total number of the DNRs by Dimnp under a specified deviation. The deviation is from 1 bp to 100 bp. By pooling together the DNRs that are pairwise identified by Danpos, we generated the DNRs data for multiple cell types for Danpos. The arrows indicated the matching percentage of the multiple cell-type DNRs between Dimnp and Danpos. In calling the DNRs, *P*-value ≤ 0.01 for Dimnp, and false discovery ratio ≤ 0.15 for Danpos. Two datasets are used. The first is a three cell-type data consisting of wild type (H4WT), mutant H3R4A and H4K20A. The second dataset consists of three mutants, H3K79A, H3S10A and H3K56A

To this end, we calculated the percentage of the matched DNRs between Dimnp and Danpos in two three-cell type datasets (Fig. 4). One dataset is for H4WT, H4R3A and H4K20A; the other is for H3K79A, H3S10A and H3K56A. Since Danpos is suitable for a two-cell type data while Dimnp is for multiple-cell type data, in order to compare the two models, we pooled the DNRs by Danpos together, and used the pooled DNRs as the DNRs data for the three cell types.

Across the strains of H4WT, H4R3A and H4K20A, 10276 DNRs were identified with Dimnp. According to our previous study [6], nucleosomes are less altered in H4R3A but are greatly altered in H4K20A in comparing with wild type. That is, mutant H4K20A is different from either H4WT or mutant H4R3A in nucleosome occupancy. Thus, it is reasonable that there is a relatively low matching percentage between the Dimnp DNRs and the Danpos DNRs of H4R3A-H4WT (Fig. 4). As expected, the Dimnp DNRs show a good matching with the pooled Danpos DNRs. The matching percentage is 60% at a 40-bp deviation (Fig. 4), which suggests that Dimnp is truly suitable for the multiple-sample comparison.

The second dataset consists of three mutants H3K79A, H3S10A and H3K56A. Nucleosomes are greatly altered in each mutant compared with wild type [6]. Dimnp identified a small number of DNRs (2536) across the three mutants. Importantly, more than 70% of the Dimnp DNRs overlap with the pooled Danpos DNRs at a deviation of ≥40 bp (indicated with an arrow in Fig. 4), showing a good matching. A small matching percentage is observed between the Dimnp DNRs (*n* = 3) and the Danpos DNRs (*n* = 2) (Fig. 4), indicating a difference between three-cell type comparison and two-cell type comparison in this case.

Taken together, we observed a matching percentage of more than 60% between Dimnp and Danpos (pooled) under a deviation of 40 bp (Fig. 4), indicating that Dimnp has capacity of detecting the differential regions across multiple cell types at one-time computation.

### An application of Dimnp

**Fig. 5:**
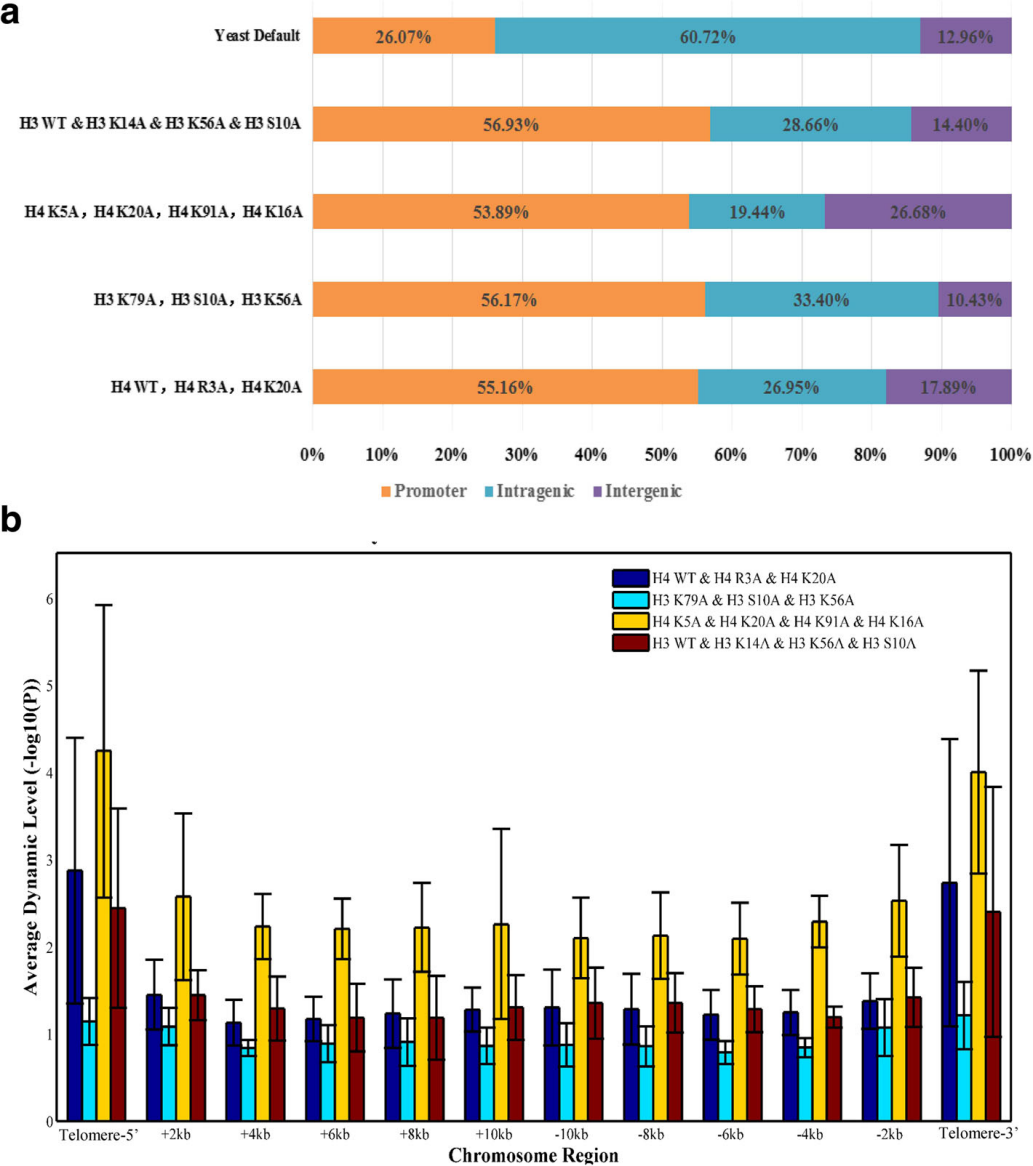
An application of Dimnp in analyzing the DNRs in histone mutation strains. **a**, Promoters enrich the DNRs upon mutations at the modifiable histone residues. Shown are the distributions of the DNRs identified by Dimnp in four sets of multiple cell types. Shown at the top panel is the percentages of promoter (−0.5 k bp ~ +0.3 k bp), intragenic region (+0.3 k bp ~ transcription termination site), and intergenic region (other region) to the genome. **b**, Average *P*-value (−log10) near the telomeres in the four sets of the multiple cell-type comparisons

We applied Dimnp to identify the DNRs in four groups of multiple cell-type datasets. The DNRs distribution indicates that more than 50% of the DNRs are located at promoters (−0.5 k bp ~ +0.3 k bp, relative to TSSs) (Fig. 5a). This percent is far greater than the percent of the promoter length in yeast genome. In human stem cells, nucleosomes are altered at key regulatory regions during cell differentiation and reprogramming, especially at enhancers [9]. In *Saccharomyces cerevisiae*, the major regulatory sites are at promoters, thus nucleosomes are altered at promoters upon the event of histone residues muation. Enrichmment analysis suggested that the nucleosomes are highly altered in genes that associate “Ras GTPase activator activity” among the mutants of histone H4 (Additional file 2: Figure S4 A and B). In the mutants of histone H3, nucleosomes are alterred in ribosome genes and sulfur amino acid metabolism-related genes (Additional file 2: Figure S4 C and D).

Another finding is that telomeres also enrich the DNRs (Fig. 5b). The results are highly consistent to our previous results [6]. This further demonstrates the well performance of Dimnp.

## Discussion

In order to identify the differential nucleosome regions in more than two cell types, we developed a Chi-squared test-based model (Dimnp). We tested the feasibility and performance of Dimnp on the datasets of sequenced reads in mutant strains of *Saccharomyces cerevisiae*. The results indicated that Dimnp has the capacity of identifying the differential regions in multiple cell types. And we provided the software packages covering the computation procedures of normalizing reads count, profiling and calling the DNRs. The output files include *P*-value and FDR for each DNR.

Dimnp does not provide a pairwise comparison after identifying the DNRs in multiple cell types. But this can be realized by running Dimnp more times.

## Conclusions

Nucleosome plays a role in gene regulation and shows dynamics among different cell types. Identifying DNRs is an important task in studying the gradual change in development and cancer, because this involves a multiple-sample (−cell type) comparison. Here, we proposed a Chi-squared test-based approach, Dimnp, to identify DNRs in multiple cell types. On the datasets of sequencing reads in yeast mutant strains, we demonstrated that Dimnp has a good capacity in comparing multiple cell types. Dimnp is able to identify all the DNRs that are identified by two-sample method Danpos. With Dimnp, we revealed that promoters and telomeres enrich more of the DNRs. Besides, we provided software package for Dimnp. The package includes the modules of both calling nucleosome occupancy and identifying DNRs, and can be easily used in analysis of nucleosome occupancy variation.

## Additional files

Additional file 1: Source codes for Dimnp. (RAR 406 kb)
Additional file 2: Figure S1. Nucleosome occupancy profiles in the vicinity of transcription start sites (TSSs) for 5419 genes in 22 mutant strains. **Figure S2.** A sample of identification of differential nucleosome regions (DNRs) with local and global background correction methods, respectively. **Figure S3.** A, Shown are reads count profiles which are normalized with the windows with a different width. B, Shown is average of coefficient of variation (CV) in each window against the width of window in the four mutant strains. The result suggests the background correction with a small window (<200 bp) will eliminate variation of nucleosome. **Figure S4.** An enrichment analysis for the nucleosome-dynamic genes. A gene with a differential nucleosome region is regarded as a nucleosome-dynamic gene. (PDF 346 kb)
Additional file 3: Data of differential nucleosome regions (DNRs) identified by Dimnp for yeast chromosome 1 in two sets of multiple-cell type comparison. One is for H4WT, H4R3A and H4K20A; the other is H4K5A, H4K20A, H4K91A and H4K16A. (XLS 86 kb)

## Abbreviations

AUC: Area under the curve
Danpos: Dynamic analysis of nucleosome position and occupancy by sequencing
Dimnp: Differential identification in multiple (*n* ≥ 3) samples
DNRs: Differential nucleosome regions
FDR: False discovery ratio
H4K20A: H4K5A, H3K79A, H3S10A, H3K56A, H4K91A and H4K16A represent the mutant strains. The abbreviation is similar as H4R3A
H4R3A: Mutant strain in which arginine (R) 3 of histone H4 was mutated into alanine (A)
H4WT: Wild type of *Saccharomyces cerevisiae*
ROC: Receiver operating characteristic
