# Supplementary material for: An approach of identifying differential nucleosome regions in multiple samples
Source: BMC Genomics. 2017 Feb 7;18:135. doi: 10.1186/s12864-017-3541-9 (PMC5297132; doi:10.1186/s12864-017-3541-9)
Supplement: Additional file 2: Figure S1. — Nucleosome occupancy profiles in the vicinity of transcription start sites (TSSs) for 5419 genes in 22 mutant strains. Figure S2. A sample of identification of differential nucleosome regions (DNRs) with local and global background correction methods, respectively. Figure S3. A, Shown are reads count profiles which are normalized with the windows with a different width. B, Shown is average of coefficient of variation (CV) in each window against the width of window in the four mutant strains. The result suggests the background correction with a small window (<200 bp) will eliminate variation of nucleosome. Figure S4. An enrichment analysis for the nucleosome-dynamic genes. A gene with a differential nucleosome region is regarded as a nucleosome-dynamic gene. (PDF 346 kb) [file 12864_2017_3541_MOESM2_ESM.pdf]

Supplementary 2

Figure legends

Figure S1 Nucleosome occupancy profiles in the vicinity of transcription start sites (TSSs) for 5419 genes in 22 mutant strains of *Saccharomyces cerevisia*.

Figure S2 A sample of identification of differential nucleosome regions (DNRs) with local and global background correction methods, respectively.

Figure S3 A, Shown are reads count profiles which are normalized with the windows with a different width. B, Shown is average of coefficient of variation (CV) in each window against the width of window in four mutant strains. The result suggests the background correction with a small window (< 200 bp) will eliminate variation of nucleosome.

Figure S4 An enrichment analysis for the nucleosome-dynamic genes.

Figure S1

Nucleosome occupancy profiles in the vicinity of transcription start sites (TSSs) for 5419 genes in 22 mutant strains of *Saccharomyces cerevisia*.

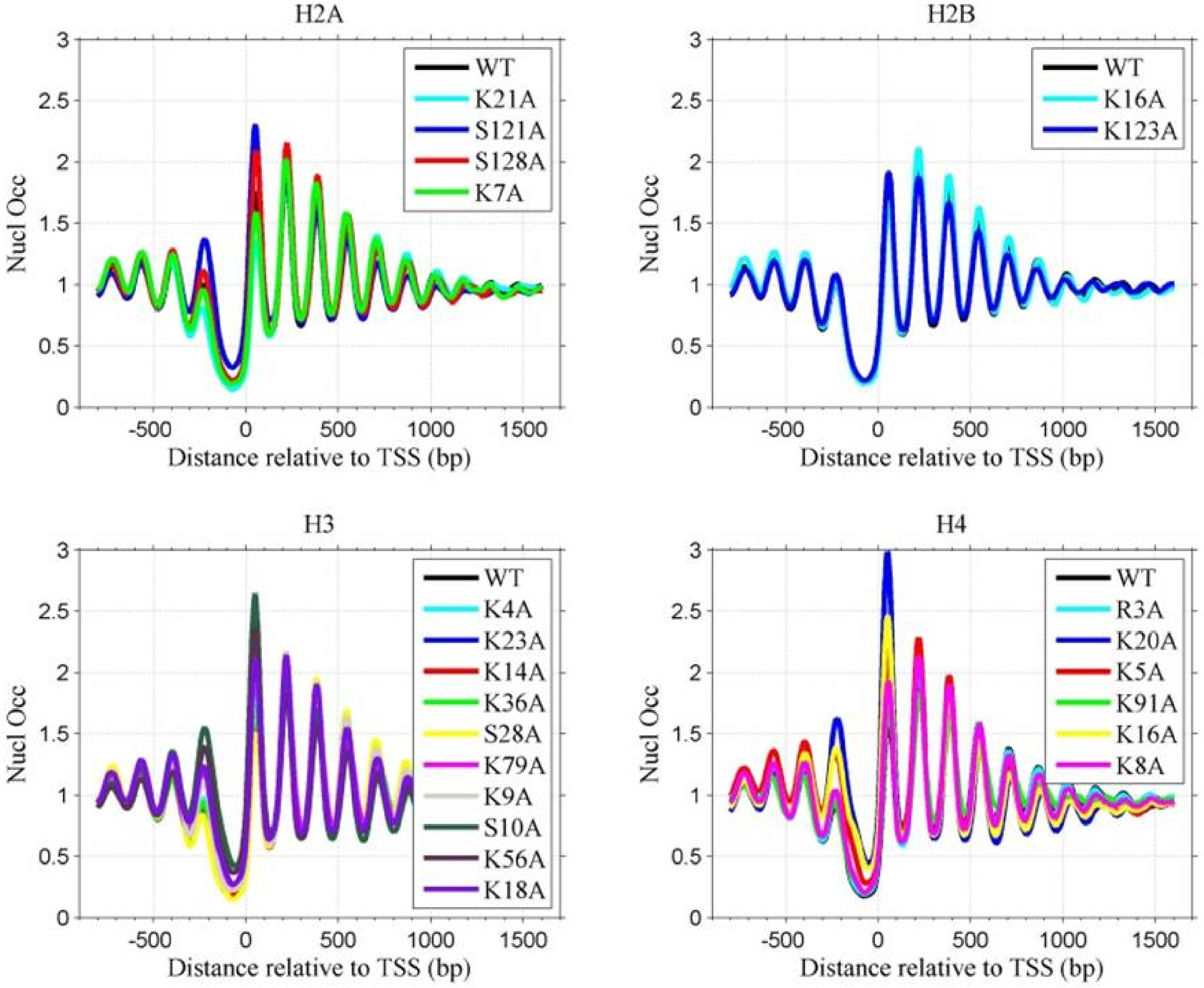

Figure S2

A sample of identification of differential nucleosome regions (DNRs) with local and global background correction methods, respectively.

Top panel indicates gene information at the genomic locus. The Second Panel is nucleosome occupancy profiles for three type of yeast strains, wild type (H4WT) and two mutants (H4R3A and H4k20A). The third panel indicates the DNRs with global back correction. The bottom panel shows the DNRs with a 10^4 bp-window local background correction. The false discovery ratio of P-value cutoff is also indicated.

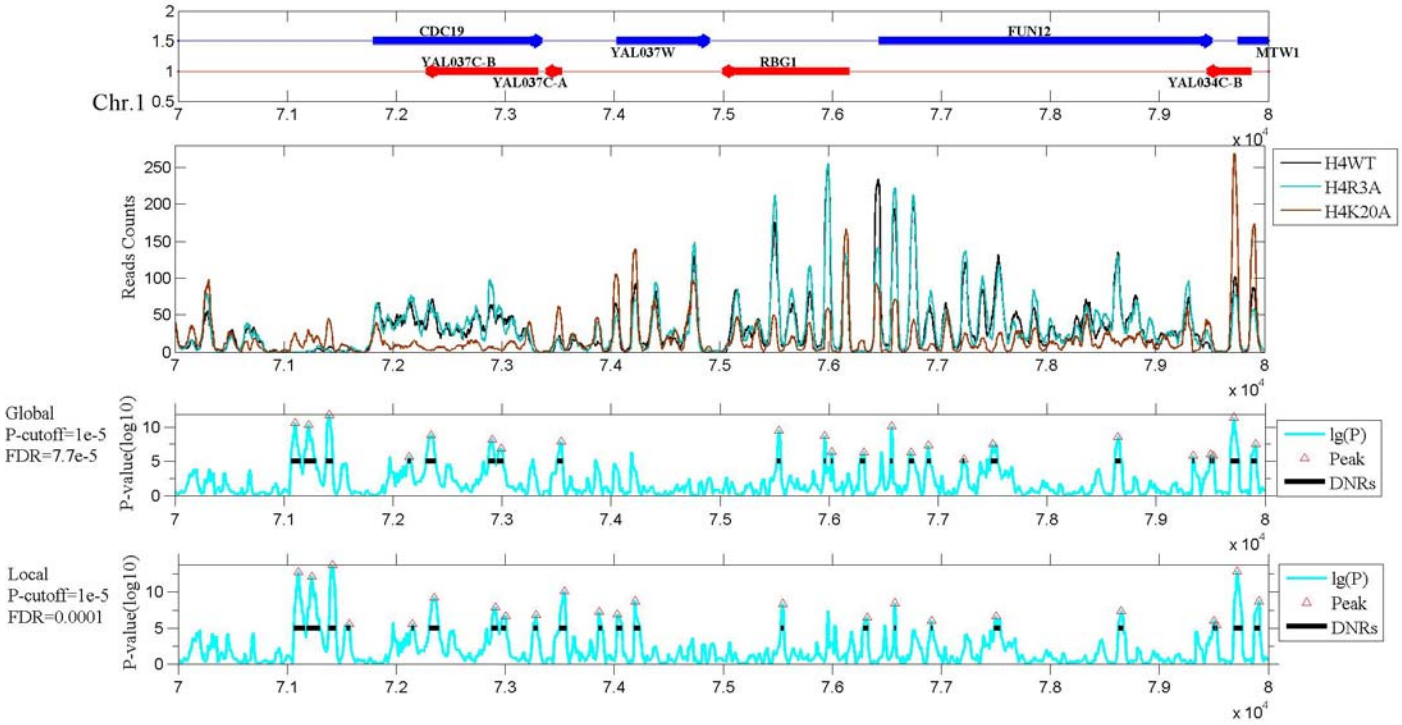

Figure S3

A, Shown are reads count profiles which are normalized with the windows with a different width;

B, Shown is average of the coefficient of variation (CV) in each window against the width of window in four mutant strains. The result suggests the background correction with a small window (< 200 bp) will eliminate variation of nucleosome.

A

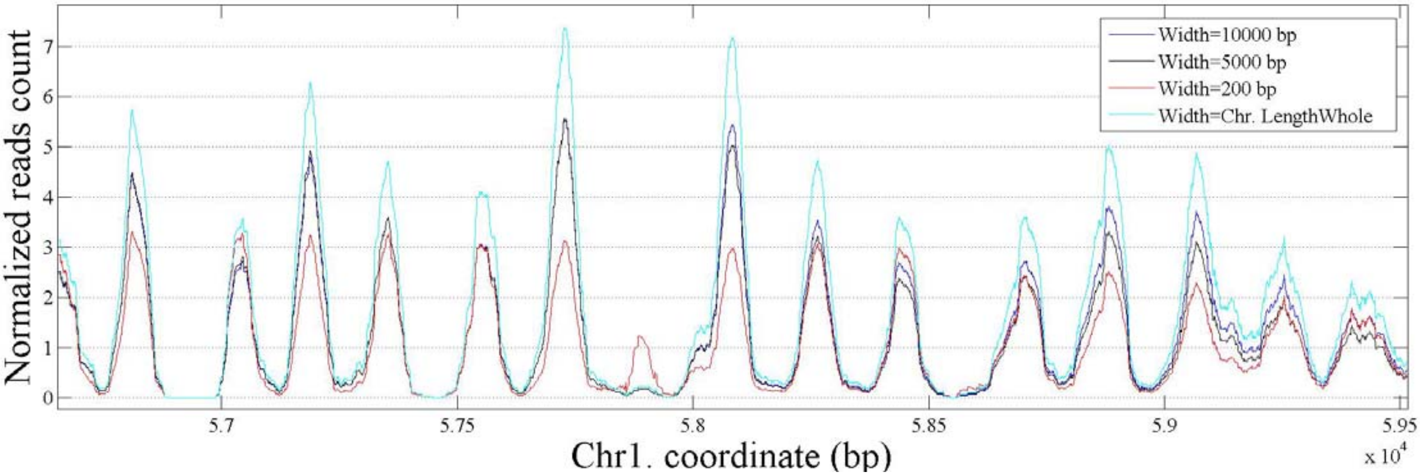

B

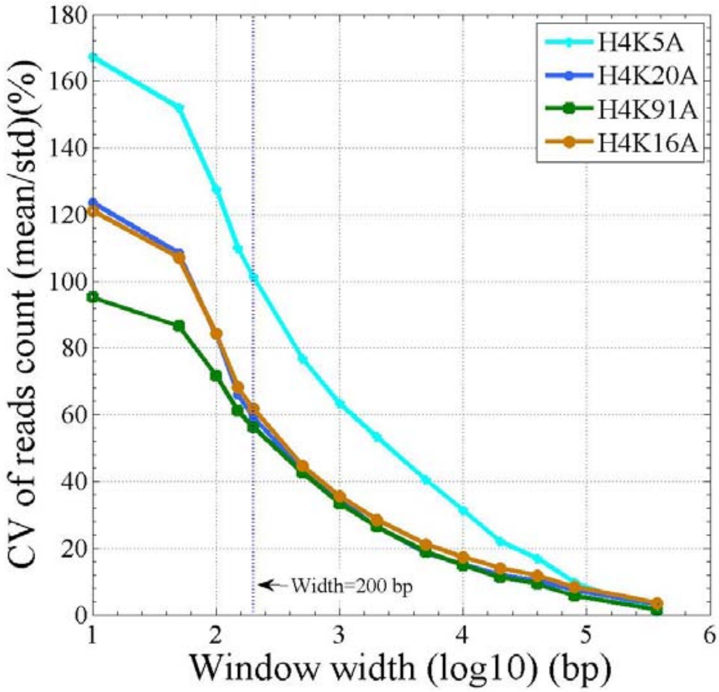

Figure S4

An enrichment analysis for the nucleosome-dynamic genes

A

Gene number: 348

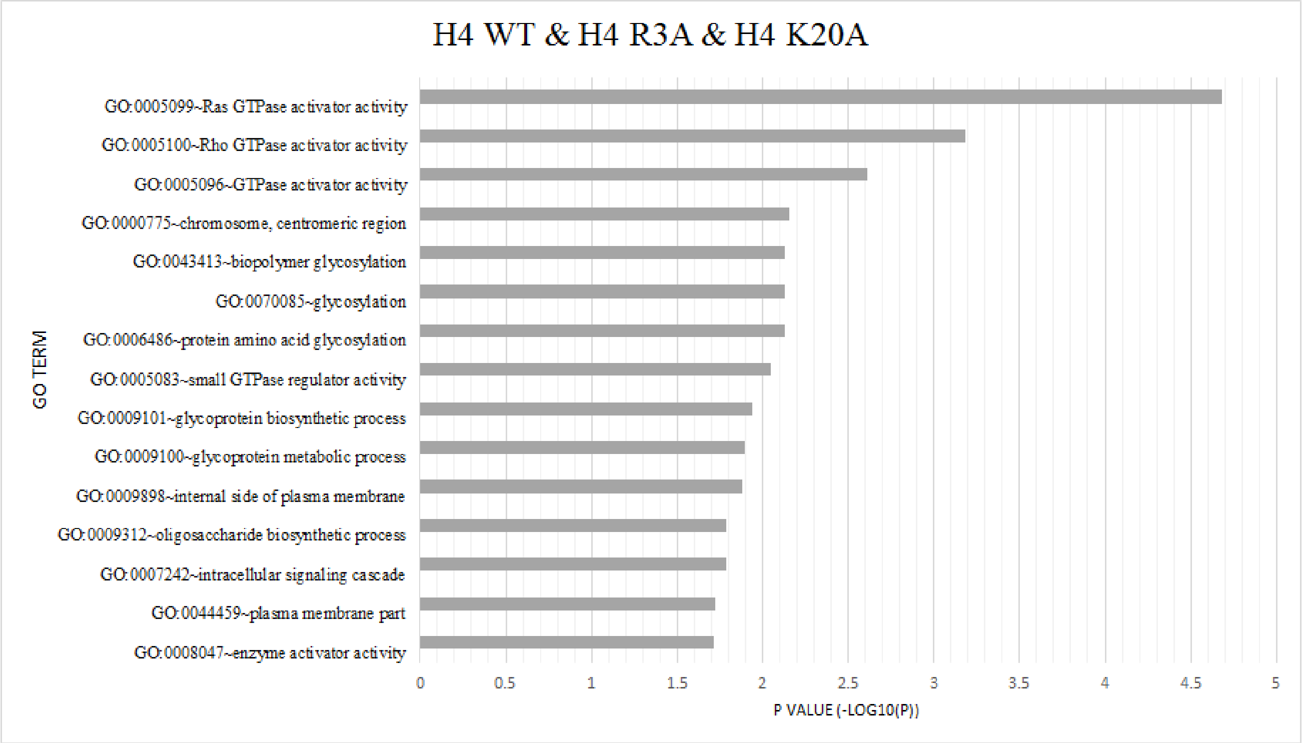

B

Gene number: 448

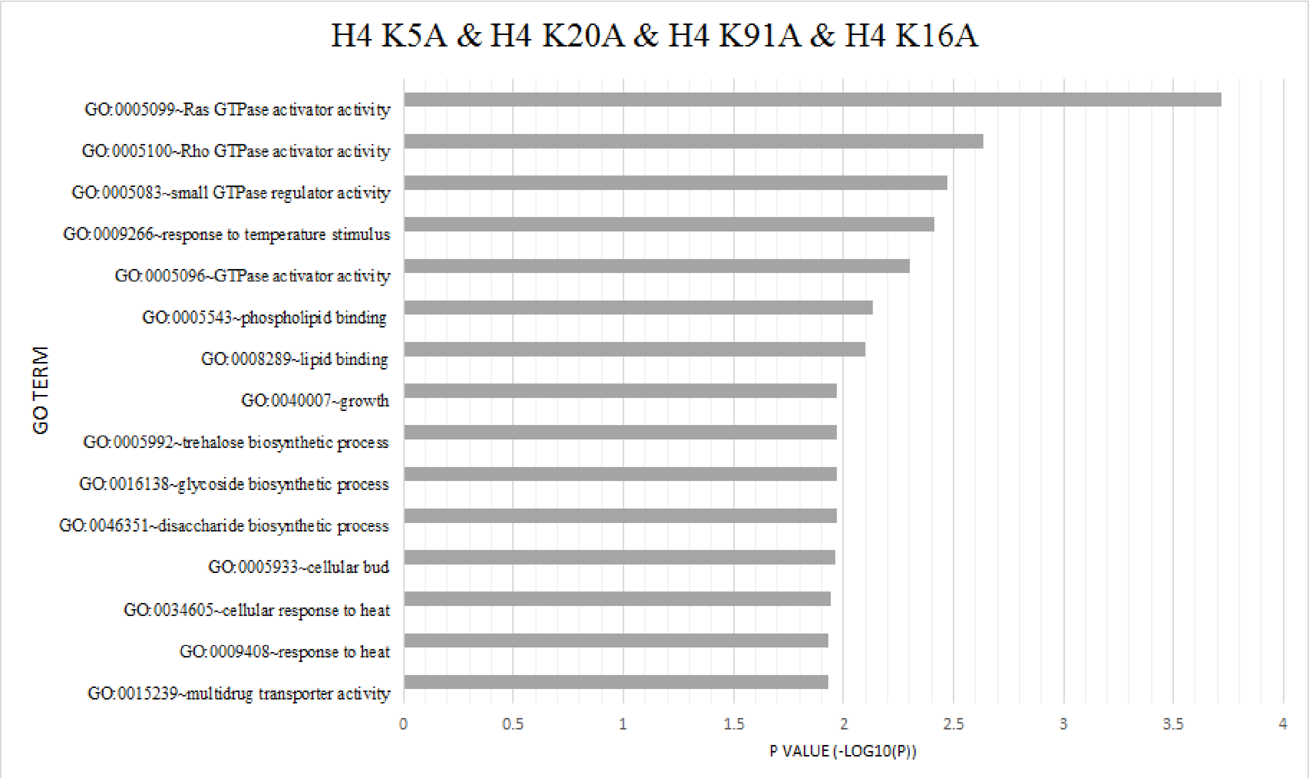

C

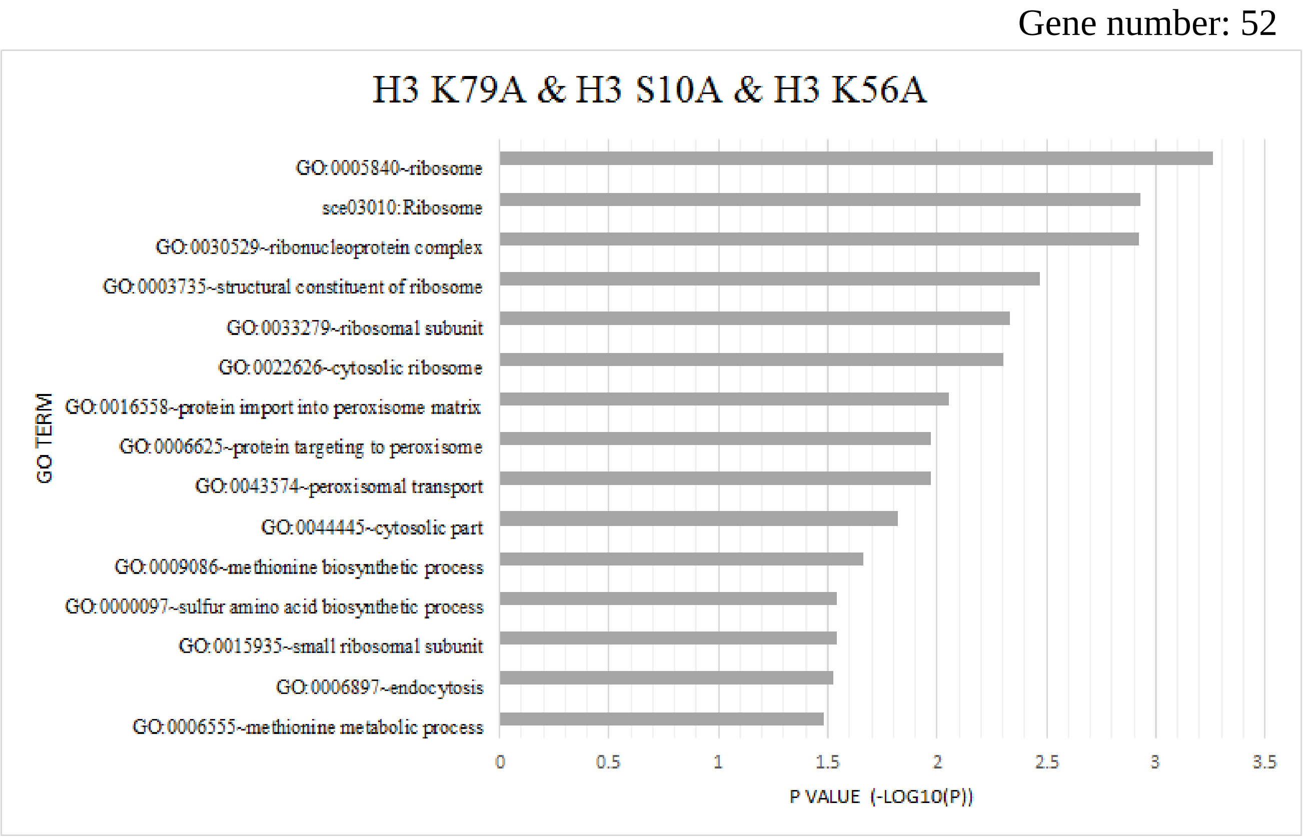
Gene number: 52
H3 K79A & H3 S10A & H3 K56A
GO:0005840~ribosome
sce03010:Ribosome
GO:0030529~ribonucleoprotein complex
GO:0003735~structural constituent of ribosome
GO:0033279~ribosomal subunit
GO:0022626~cytosolic ribosome
GO:0016558~protein import into peroxisome matrix
GO:0006625~protein targeting to peroxisome
GO:0043574~peroxisomal transport
GO:0044445~cytosolic part
GO:0009086~methionine biosynthetic process
GO:0000097~sulfur amino acid biosynthetic process
GO:0015935~small ribosomal subunit
GO:0006897~endocytosis
GO:0006555~methionine metabolic process
GO TERM
0
0.5
1
1.5
2
2.5
3
3.5
P VALUE (-LOG10(P))

D

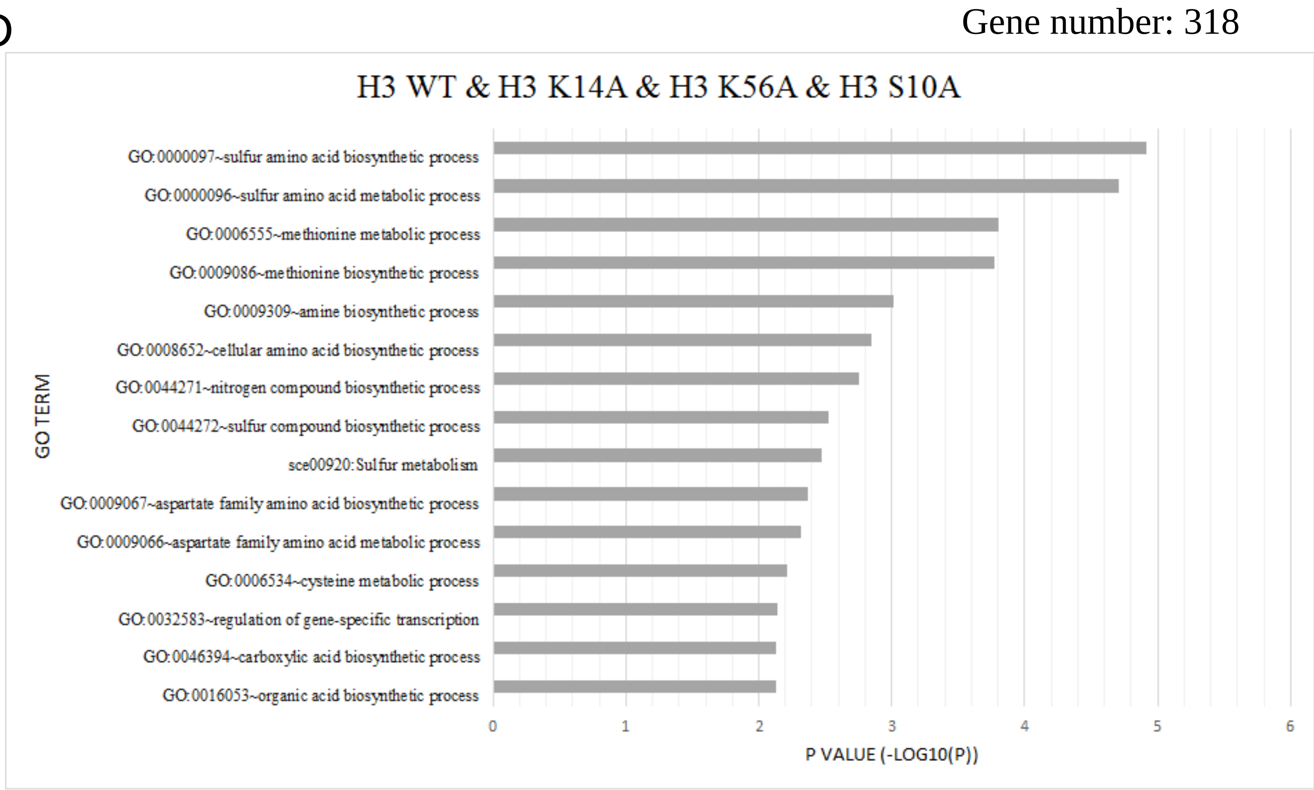
Gene number: 318
H3 WT & H3 K14A & H3 K56A & H3 S10A
GO:0000097~sulfur amino acid biosynthetic process
GO:0000096~sulfur amino acid metabolic process
GO:0006555~methionine metabolic process
GO:0009086~methionine biosynthetic process
GO:0009309~amine biosynthetic process
GO:0008652~cellular amino acid biosynthetic process
GO:0044271~nitrogen compound biosynthetic process
GO:0044272~sulfur compound biosynthetic process
sce00920:Sulfur metabolism
GO:0009067~aspartate family amino acid biosynthetic process
GO:0009066~aspartate family amino acid metabolic process
GO:0006534~cysteine metabolic process
GO:0032583~regulation of gene-specific transcription
GO:0046394~carboxylic acid biosynthetic process
GO:0016053~organic acid biosynthetic process
GO TERM
0
1
2
3
4
5
6
P VALUE (-LOG10(P))
